# Heat Stress and Betaine Affect Lipolysis in Pig Adipose Tissue Explants

**DOI:** 10.3390/ani15192845

**Published:** 2025-09-29

**Authors:** Zaira Pardo, Manuel Lachica, Rosa Nieto, Isabel Seiquer, Ignacio Fernández-Fígares

**Affiliations:** 1 Animal Nutrition Program, Institute of Agrifood Research and Technology (IRTA), 43120 Constantí, Spain; zaira.pardo@irta.cat; 2 Department of Nutrition and Sustainable Animal Production, Estación Experimental del Zaidín, Consejo Superior de Investigaciones Científicas, 18014 Granada, Spain; manuel.lachica@eez.csic.es (M.L.); rosa.nieto@eez.csic.es (R.N.); isabel.seiquer@eez.csic.es (I.S.)

**Keywords:** thermal stress, swine, glycerol release, fat, trimethyl glycine

## Abstract

Heat stress (HS) is an environmental variable challenging pig production worldwide. It may alter carcass composition by increasing fat deposition but the mechanisms are poorly understood. Betaine is a metabolic modifier able to decrease carcass fat in pigs, but the effects of betaine on lipolysis under high temperatures are not known. Our goal was to determine a potential effect of betaine on lipolysis in the fat tissue of Iberian pigs under thermoneutral (TN) or HS conditions. Eight pigs (89 kg BW) were used to obtain explants from dorsal subcutaneous adipose tissue. Explants were cultivated under TN (37 °C) or HS conditions (41.5 °C) for 1.5 h to study direct effects of HS on acute lipolysis. Treatments included control and betaine addition (200 µM). Indirect effects of HS were examined using the culture medium amended with 1 μM isoproterenol ±10 nM insulin in the presence or absence of betaine and HS. Media glycerol was measured at the end of the incubations as a proxy of lipolysis. HS decreased basal lipolysis (−47%; *p* < 0.001) but increased isoproterenol-stimulated (a β-agonist) lipolysis (+31%; *p* < 0.01). Betaine increased non-stimulated lipolysis both under TN and HS conditions (73%; *p* < 0.001). As expected, isoproterenol augmented lipolysis both in TN (2714%; *p* < 0.001) and HS (3589%; *p* < 0.001) conditions. The addition of insulin to lipolysis-stimulated explants analogously diminished glycerol release in TN (−39%; *p* < 0.001) and HS (−50%; *p* < 0.001) conditions. In conclusion, HS reduced non-stimulated lipolysis in Iberian pigs in line with increased adipose tissue accretion, while betaine partly increased lipolysis, counteracting the effects of HS. Betaine supplementation could be a strategy to counteract HS-induced fattening in pigs.

## Simple Summary

Heat stress (HS) is a major environmental factor that compromises pig production efficiency. Also, HS can modify carcass composition and value by augmenting fat and/or reducing lean deposition. The effects of HS on carcass composition are not constant, depending, among others, on the intensity and duration of HS, the physiological stage of the pig or feeding management. Previous studies have demonstrated that HS is associated with alterations in energy metabolism. It is also known that betaine is a growth promoter able to alter nutrient partitioning in pigs. In this study, the effect of HS on lipolysis in adipose tissue explants from Iberian pigs was investigated. Additionally, the regulation of lipolysis and the effect of betaine were investigated.

## 1. Introduction

Heat stress (HS) is a major environmental factor affecting pig metabolism, health and welfare, and has a negative impact on growth, causing large economic losses [1]. This is considerably relevant particularly in extensive systems of animal production as the frequency of heat spells is expected to increase as a consequence of climate change. Pig performance is affected under high-temperature episodes because feed intake is reduced to decrease heat production [2]. Furthermore, pigs are very sensitive to HS because the ability to dissipate heat is impaired due to scattered sweat glands and a thick subcutaneous fat depth [3]. Iberian pigs comprise a native breed of the Iberian Peninsula with an abundant subcutaneous layer of fat and reduced feed intake (20%) under persistent HS conditions [4]. It is consistently reported that HS attenuates fat mobilization, which can potentially aggravate carcass fatness when weight is increased [5,6,7]. Indeed, HS leads to increased expression of genes involved in fatty acid uptake and triglycerides synthesis (e.g., FAS, LPL, GLUT4, PCK1, and GK) [8], and elevates lipogenic pathways while suppressing fatty acid oxidation according to a metabolomics approach [9].

Adipose tissue explants have been used to study the lipolytic response under various conditions [10,11]. Indeed, we have shown that betaine increased short-term lipolysis but had no effect on long-term lipolysis [10]. Moreover, Dufau et al. [11] described the usefulness of adipose tissue explants to investigate lipolytic response, inflammation or angiogenesis in an extensive review on in vivo and ex vivo models of adipocytes. Nevertheless, the mechanisms by which HS influences lipolysis remain unclear.

Betaine or trimethyl glycine is a choline derivative utilized by animals primarily as an osmolyte [12], and as a methyl donor for the re-methylation of homocysteine to methionine [13]. Betaine is able to promote growth in pigs under thermoneutral conditions [14,15]. As a matter of interest, the positive effects of dietary betaine on average daily gain and gain to feed ratio seem to be more apparent under heat stress or nutrient restriction [16,17,18,19]. Nevertheless, in a previous experiment we were not able to detect an effect of dietary betaine on the metabolic heat production of Iberian pigs under chronic heat stress [20], in contrast with a slight decrease in heat production in conventional pigs [21]. We have already shown that dietary betaine decreased fat deposition while increasing carcass lean in conventional pigs [22]. However, dietary betaine did not produce significant changes in growth performance, body composition [23] and yield of lean cuts [24] in Iberian pigs. Interestingly, biochemical and hormone profiles could partially explain an increase in lean deposition in pigs fed with betaine and CLA diet [25]. Betaine decreased portal-drained viscera heat production in Iberian pigs, augmenting the energy available for peripheral tissues [26]. However, it must also be considered how energy is divided between fat and protein. Although the mechanisms explaining the reduction in carcass fat in pigs fed betaine are poorly understood, the inhibition of lipogenesis [27] and stimulation of lipolysis should play a role. In a previous experiment, we have shown that betaine produced a biphasic response so that lipolysis was increased in acute conditions but not in chronic conditions using explants from Iberian pigs [10]. Moreover, although long-term heat stress deranged β-cell function estimated through the homeostasis model assessment index, betaine partially reversed the effect [4]. Further details on the use of dietary betaine in humans and different farm species as well as molecular mechanisms can be found in a recent comprehensive review [28]. With this background, the aim of the present work was to determine the effect of HS and betaine supplementation on lipolysis in pig adipose tissue explants.

## 2. Materials and Methods

### 2.1. Adipose Tissue Isolation and Culture

Adipose tissue was isolated from eight Iberian barrows of 89 ± 4.2 kg BW purchased from Sanchez Romero Carvajal Jabugo S.A. (Puerto de Santa María, Cádiz, Spain). Pigs were allocated in a thermoregulated room (21 ± 1.0 °C) and fed with a standard barley, corn-soybean meal diet (146 g crude protein/kg, 8.9 g lysine/kg and 16.6 MJ metabolizable energy/kg) supplemented with essential amino acids to achieve an adequate amino acid profile [29]. Pigs were slaughtered by exsanguination after electrical stunning. The process for isolation and culture was carried out according to Fernández-Fígares et al. [10] and Ramsay and Richards [30]. In brief: immediately after slaughter, dorsal subcutaneous adipose tissue samples were taken from the neck because it is an area free of muscle tissue and easily accessible. Afterwards, the tissue was cut into pieces (10 × 40 mm), placed in Hanks buffer in beakers (37 °C and pH 7.4) to clean up any residual blood and covered with parafilm to avoid any contamination during transport to the cell culture laboratory. In the laboratory, fat tissue was transported in fresh Hanks buffer (37 °C and pH 7.4), cleaned of muscle and cut into 10 mm cubes under a laminar flow hood. Adipose tissue explants were then prepared by cutting adipose tissue cubes using a Stadie-Riggs microtome. The weight of the adipose tissue explants was approximately 100 mg. Each of the 400 µM thick slices of adipose tissue were washed twice with Hanks buffer, dried of excess liquid, weighed and transferred to six-well plates. Each well of the plate contained 2 mL of basal medium (DMEM/F12 (50:50), 25 Mm HEPES, gentamycin, 0.5% bovine serum albumin, amphotericin B and penicillin-streptomycin) and the slices were incubated (Forma Scientific Inc., Marjetta, OH, USA; 37 °C, 5% CO_2_) for 1.5 h to wash glycerol and fatty acids that might have been released during the isolation.

**Figure 1:**
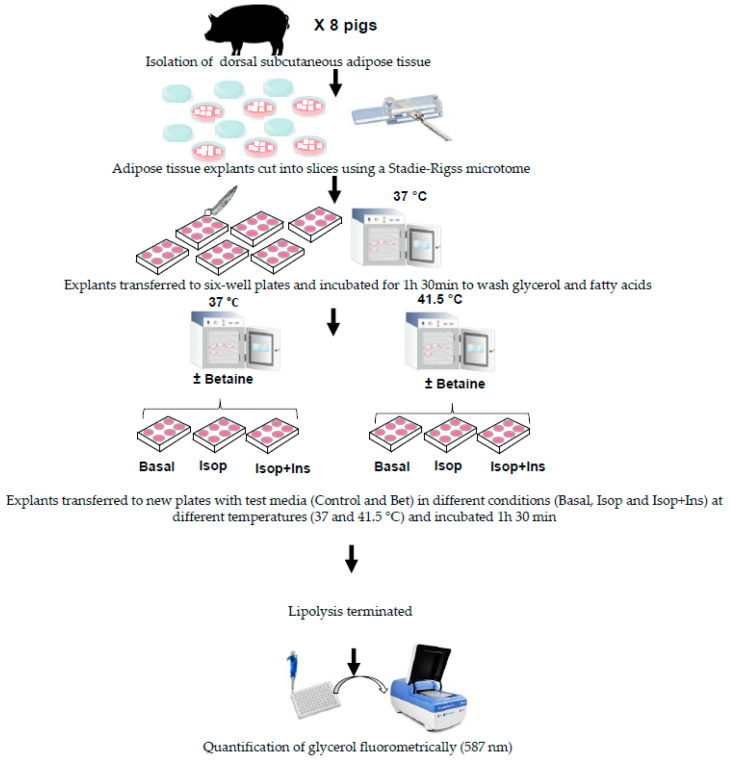
Diagram of the experimental design. Isop: Isoproterenol; Isop + Ins: Isoproterenol plus insulin.

For each pig, replicates of tissue slices were incubated with one of the test mediums: basal medium (Control) and basal medium amended with betaine (200 µM; Bet; Aldrich #21,906-1, 98% purity; Tres Cantos, Madrid, Spain) and exposed at TN (37 °C) or HS (41.5 °C) during 1 h 30 min. The incubation time was chosen to study the effects of betaine and HS on acute lipolysis. The optimal temperature for adipocyte culture in pigs is 37 °C and it is routinely used in adipocytes and adipose tissue explants (e.g., [10,31]). To study the effect of heat stress on pig pre-adipocytes differentiation and fatty acid composition temperatures between 41 and 42 °C were used [9,31]. The first assay served to estimate the direct effect of betaine on acute basal lipolysis at both thermal conditions. Furthermore, a second assay was run to analyze the ability of betaine and HS to alter the suppression of isoproterenol-stimulated lipolysis elicited by insulin. For this purpose, replicates of tissue slices were exposed to Control and Bet media added with 1 μM isoproterenol (#I2760, Sigma-Aldrich, St. Louis, MO, USA) or 1 μM isoproterenol + 10 nM insulin (#I1882, Sigma-Aldrich, St. Louis, MO, USA) and incubated at both temperatures for 1 h 30 min. The preferred β-agonist was isoproterenol due to its significant response in swine adipose tissue [10]. Concentrated solutions of isoproterenol and bovine insulin were previously prepared at 1000× in milli Q water and 0.01 N ClH, respectively, and sterile filtered through a 0.22 μm membrane. All the assays were run in triplicate, and eight pigs were used. The experiment design is depicted in Figure 1.

After incubation, the medium was removed, and lipolysis was evaluated by determining glycerol release. Medium samples (1 mL) were added with 0.1 mL 30% HClO_4_ and centrifuged at 13,000× *g* for 5 min. Then, supernatants were neutralized with 1 N KOH and frozen for later analysis of glycerol content. Glycerol is released during the hydrolysis of triglycerides correlating with free fatty acids release. Glycerol was measured using a commercial kit, following the manufacturer directions (#MAK117, Sigma-Aldrich, St. Louis, MO, USA). Briefly, the kit quantified glycerol concentration by a coupled enzymatic assay involving glycerol phosphate oxidase and glycerol kinase, generating a product that can be quantified fluorometrically (λ_ex_ = 535/λ_em_ = 587 nm) and that is proportional to the glycerol present in the medium. Samples were analyzed in duplicate.

### 2.2. Statistical Analysis

The present experiment was analyzed as a linear model with several factors and their interactions. Specifically, it was µ_ijklm = Ai + Tj + Bk + Ll + (TB)jk + (TL)jl + (BL)kl + (TBL)jkl + (ATBL)ijkl + eijklm, where Ai corresponds to the effect of the animal (i = 1, 2, 3, 4, 5, 6, 7, 8); Tj, the effect of temperature (j = 1, 2); Bk, the effect of betaine level (k = 1, 2); Ll, the effect of lipolysis stimulation (l = 1, 2, 3); and m, the effect of the plate (1, 2, 3). (ATBL)ijkl represents the interaction between experimental factors, including the animal. The null hypothesis was that there were no differences in lipolysis between treatments. All hypothesis testing was two-tailed analyzed with a significance level, α, 0.05. The statistical power of the experiment was set up at β = 0.80 to detect significant differences between treatments. When a significant interaction was found, post hoc comparisons between treatment means were performed using the Least Significant Difference (LSD) test. Data were analyzed using the PROC MIXED procedure of SAS (1989). All statistical analyses were carried out using SAS (version 9.4, SAS Institute Inc., Cary, NC, USA).

## 3. Results

### 3.1. The Effect of Heat Stress on Acute Lipolysis

**Figure 2:**
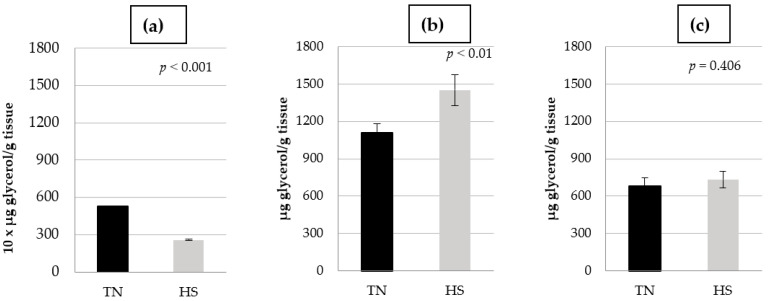
Glycerol release in thermoneutral (TN) or heat stress (HS) conditions: (**a**) basal lipolysis, (**b**) isoproterenol-stimulated lipolysis, (**c**) isoproterenol + insulin lipolysis.

The effect of HS on glycerol release is shown in Figure 2. An interaction between temperature and lipolysis conditions was found (*p* < 0.001). HS decreased lipolysis under basal conditions (47%; *p* < 0.001). Nevertheless, HS increased isoproterenol-stimulated lipolysis (31.1%; *p* < 0.01) and did not affect (*p* = 0.406) insulin suppression of isoproterenol-stimulated lipolysis.

### 3.2. The Effect of Betaine on Acute Lipolysis

**Figure 3:**
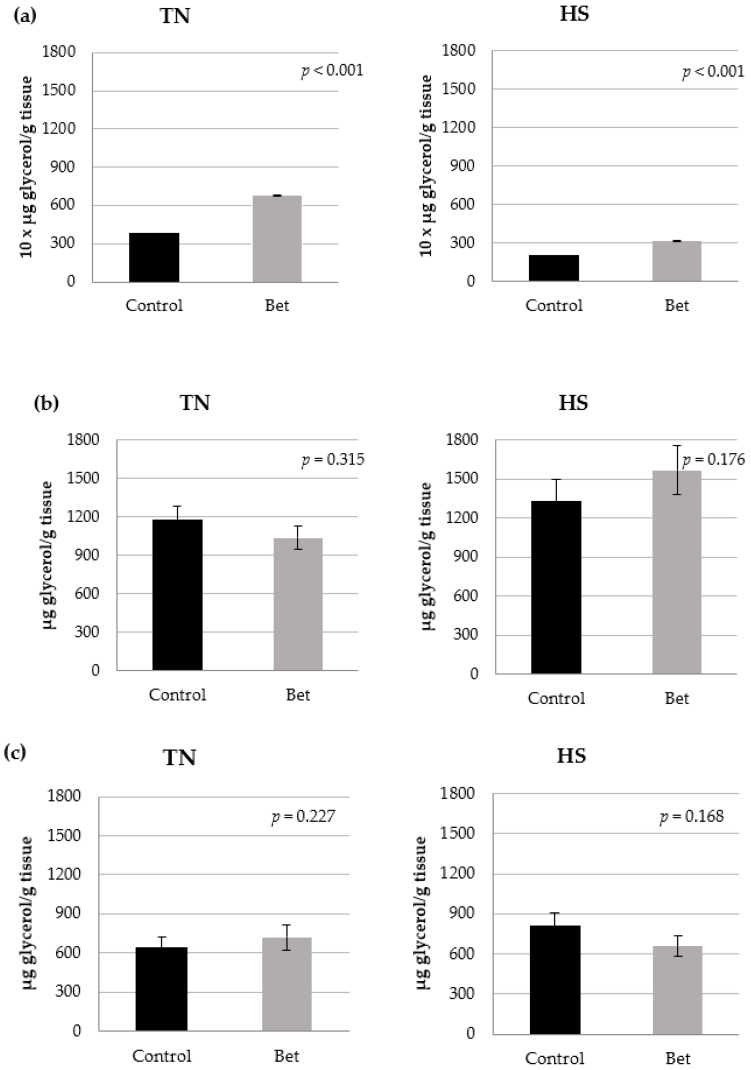
Glycerol release in response to betaine (Bet; 1 mM) exposure in thermoneutral (TN) and heat stress (HS) conditions: (**a**) basal lipolysis, (**b**) isoproterenol-stimulated lipolysis, (**c**) isoproterenol + insulin lipolysis.

The effects of betaine on lipolysis are shown in Figure 3. An interaction between betaine addition and lipolysis conditions was found (*p* < 0.001). Betaine increased lipolysis in basal conditions both under TN and HS (81 and 57%, respectively; *p* < 0.001; Figure 3a). Nevertheless, no effect of betaine could be detected when lipolysis was stimulated by isoproterenol both in TN and HS conditions (*p* > 0.10; Figure 3b). Furthermore, betaine did not affect lipolysis both in TN and HS conditions in the presence of isoproterenol plus insulin (*p* > 0.10; Figure 3c).

### 3.3. The Regulation of Acute Lipolysis by Isoproterenol and Insulin

**Figure 4:**
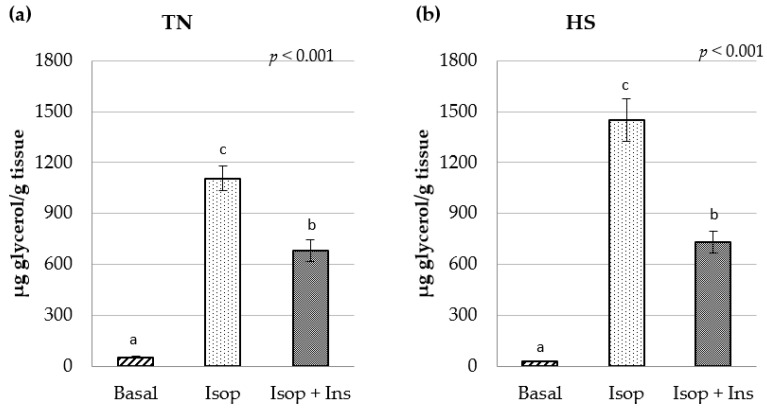
Glycerol release after exposure (1.5 h) to basal media, isoproterenol (Isop; 1 µM) and isoproterenol + insulin (Isop + Ins; 10 nM) under (**a**) thermoneutral (TN) and (**b**) heat stress (HS) conditions. Different letters indicate significant differences.

The effects on lipolysis of isoproterenol and insulin are depicted in Figure 4. Isoproterenol stimulated lipolysis (*p* < 0.001) both in TN and HS conditions relative to basal medium (by 3152 and 1696%, respectively; *p* < 0.001). However, the addition of insulin (10 nM) to the medium reduced the isoproterenol-stimulated lipolytic response in TN and HS conditions (by 39 and 50%, respectively; *p* < 0.001).

## 4. Discussion

HS represents a significant environmental challenge in pig production, particularly in regions where high temperatures compromise animal welfare, productivity and carcass quality [20]. Conventional pigs under HS decreased plasma non-esterified fatty acids despite diminished feed intake, especially when compared with pair-fed, TN controls [32].

Moreover, HS increases the expression of genes involved in fatty acid uptake and triglycerides synthesis in Yorkshire × Landrace adipocytes in vitro [8], thus increasing fat deposition [33,34] and negatively affecting meat quality [5]. Iberian pigs have remarkable metabolic and phenotypic differences with lean cosmopolitan crossbreeds due to their thrifty genotype [35], enabling efficient energy storage on refeeding after periods of food shortage for the preparation of subsequent hunger [36,37]. Indeed, noticeable differences in meat quality [38] and composition [39] have been reported between Iberian and cosmopolitan pigs.

In the case of the Iberian pig, HS could be a major challenge as well, as it is characterized by a thick subcutaneous fat layer increasing insulation compared with conventional breeds [40]. Iberian pigs, however, are considered rustic, resilient animals adapted to rigorous environmental conditions outdoors. Our lab has recently estimated that the upper critical temperature—the temperature above which the animal initiates thermoregulatory mechanisms to compensate HS—in growing-finishing Iberian pigs is between 28 and 30 °C [41], that is, 5 to 8 °C higher than the available data for fast-growing Western commercial breeds, showing a better adaptation of Iberian pigs to hot environments.

We have previously used ex vivo studies with porcine adipose tissue explants [10] to elucidate the possible mechanisms by which fat deposition occurs as affected by metabolic modifiers. Using the same approach, HS decreased short-term basal lipolysis in the conditions of the present experiments (Figure 2), which is consistent with in vivo studies in which HS cosmopolitan pigs showed increased fat in the carcasses due to reduced lipid mobilization [5,6,7]. Of interest, HS did not alter insulin signaling markers in adipose tissue [42]. However, a lack of adipose tissue mobilization might be the result of enhanced insulin action by other compounds. For instance, plasma lactate, which increased in a variety of HS models [43,44], mediates insulin antilipolytic effects by interacting with the G protein-coupled receptor 81 [45]. Moreover, thyroid hormones, reduced in growing pigs under HS [42] might also contribute to the lack of adipose tissue mobilization as thyroid hormones stimulate lipolysis and NEFA utilization [46]. Furthermore, HS increased the expression of genes involved in fatty acid uptake and triglycerides synthesis [8] in porcine stromovascular cells of crossbred pigs, and elevated lipogenic pathways while suppressing fatty acid oxidation in Ossabaw pigs (fatty pig derived from Iberian pigs), as evaluated through a metabolomic approach [9]. Moreover, HS diminished the expression of lipolytic genes in PIC 337 × C22/C29 pigs, leading to increased carcass fat compared to pair-fed TN controls [47]. In Iberian pigs, long-term HS augmented intramuscular fat content, and had a positive influence on lightness, drip loss and antioxidant capacity without affecting fatty acid composition [48], whereas no effect of HS on mesenteric fat (a proxy of visceral fat) was reported [4]. Furthermore, chronic HS reduced the percentage of lean cuts in Iberian pigs compared to pair-fed TN counterparts [49], which may indicate fatter carcasses under HS conditions. However, no differences in fat depth at different carcass sites were reported. No effect of long-term HS on plasma triglyceride concentration has been reported in Iberian pigs [4] and cosmopolitan breeds of different ages [31,32,50], indicating that plasma triglycerides are relatively stable during HS. With a different approach, chronic HS did not affect heat production or retained energy estimated using indirect calorimetry in Iberian pigs [20], which points out the effective adaptation of Iberian pigs to elevated temperature. Nevertheless, energy partitioning into protein and fat could fluctuate despite unaltered energy retention. Certainly, the RQ (respiratory quotient = CO_2_ production/O_2_ consumption) indicated increased lipid accretion in HS compared with pair-fed TN Iberian pigs [20]. Lipid accretion is the result of lipogenesis and lipolysis [51]. As a matter of interest, HS decreased de novo lipogenesis estimated through acetyl-CoA-carboxylase activity in backfat, leaf fat and liver of Large White × Landrace growing pigs compared to pair-fed TN counterparts [52]. In line with this, the elevated glucose concentration of Iberian pigs under chronic HS conditions could be explained by a decreased rate of glucose uptake by adipose tissue [4]. We have no further information regarding how lipogenesis is affected by HS in Iberian pigs or any other obese breed. Interestingly, malic enzyme and glucose-6-phosphate dehydrogenase were greater in Iberian than in Landrace × Large White 25 kg BW pigs, particularly in adipose tissue. Diminished adipose tissue lipolysis and subsequent fatty acid oxidation could be explicated as an evolutionary process to diminish metabolic heat production as lipolysis produces more metabolic heat than carbohydrate and protein oxidation (approximately 39.3, 15.6 and 16.7 kJ/g, respectively) [53].

Nutritional strategies to alleviate heat stress in pigs include the use of betaine (see review by Cottrell et al.) [54]. The osmotic capacity of betaine may help to deal with heat stress. Dietary betaine accumulates in pig muscle, which may augment the retention of water and decrease osmotic stress under heat stress [55]. Additionally, we have shown that betaine supplementation tends to diminish portal-drained viscera heat production [26], suggesting that betaine may spare energy that can be used by peripheral tissues, as supported by the demonstrated decrease in O_2_ consumption [26]. A decrease in fat thickness may help under heat stress conditions as it impairs heat dissipation [56]. A meta-analysis concluded that betaine decreases back fat and 10th rib fat thickness in finishing pigs [57], which would help to dissipate body heat. Betaine has been shown in other studies to have a lipolytic effect acting by several mechanisms such as the upregulation of lipolytic genes METTL3/14 and YTHDF1/2 and also the increase in the release of glycerol, the main component of fatty acids [10,58]. In the present study, betaine increased acute non-stimulated lipolysis in adipose tissue explants from Iberian pigs under TN conditions, as previously reported [10]. Furthermore, this effect was independent of environmental temperature, that is, betaine increased lipolysis under HS conditions, partly counteracting the inhibition of lipolysis elicited by HS. Betaine has also been shown to decrease the adipose tissue of growing Ningxiang pigs [59] in TN conditions. Additionally, betaine reduced de novo lipogenesis in TN by lowering the activity of fatty acid synthase while promoting fat degradation by enhancing hormone-sensitive lipase activity in adipose tissue [60]. These results are consistent with previous studies suggesting that betaine alters energy partition, reducing fat accumulation and increasing lean tissue content in pigs [22,55].

β-agonists and insulin are key regulators of lipid metabolism. We designed experiments to elucidate possible interactions of β-agonists and insulin with HS and betaine. Pioneering studies showed that catecholamines stimulate adipose tissue lipolysis in several species [61], including swine. Triglyceride hydrolysis in adipocytes is regulated by catecholamines that bind to β-adrenergic receptors to activate hormone-sensitive lipase [62]. Isoproterenol is a non-selective β-agonist with greater capacity for adipose tissue lipolysis than adrenaline and noradrenaline in pigs [63], and it has been traditionally used to study lipid metabolism under in vitro conditions (e.g., [31]). In the pig, the β1 subtype receptor represents nearly 80% of the total receptors [64,65] and seems to be the primary subtype mediating lipolysis. However, as far as we know, there is no information regarding the effect of β-agonists on lipolysis under HS conditions.

We have previously reported the stimulation of lipolysis by isoproterenol in adipose tissue of Iberian pigs under TN conditions [10]. As expected, in the present experiment, isoproterenol stimulated lipolysis in adipose tissue of Iberian pigs, although this stimulus was less patent under HS conditions (Figure 4). Interestingly, HS elicited opposite effects on lipid mobilization, depending on the presence or not of β-agonists in the media. While HS decreased basal lipolysis, glycerol release was augmented by HS when isoproterenol was added to the media (Figure 2). We have no explanation for the augmentation of glycerol release by HS under β-adrenergic stimulation.

On the other hand, betaine had no lipolytic effect under β-adrenergic stimulation during HS (Figure 3), which is surprising as dietary betaine is beneficial specifically under energy-limiting and/or stressful conditions [21]. Insulin is a powerful regulator of lipid metabolism [66], decreasing the rate of lipolysis in the adipose tissue, stimulating the synthesis of fatty acids and triacylglycerol in tissues, and lowering plasma fatty acid level. It also influences the triglyceride uptake from the blood into the muscle and adipose tissue, and decreases the fatty acid oxidation rate in the muscle and liver.

Several studies have demonstrated that HS and betaine can modify the sensitivity to insulin of different tissues. Of interest, pig adipose tissue has a limited antilipolytic response to insulin [67]. HS increased basal and stimulated circulating insulin and decreased adipose tissue mobilization in crossbred pigs [, 42] while maintaining whole-body insulin sensitivity [68]. Nevertheless, Iberian pigs subjected to a prolonged HS (30 °C for 28 days) showed no changes in insulin sensitivity through HOMA-IR (peripheral tissue resistance) and QUICKI (hepatic insulin sensitivity) [4]. This difference among studies could be due to variations in the severity of HS or the genotype of the pigs, as earlier findings indicated differences in postprandial insulin levels between lean and obese pig lines, with the latter typically exhibiting higher insulin levels [69,70].

In the present experiments, insulin blunted the lipolytic effect of HS under β-agonist stimulation (Figure 2), suggesting that HS increased the antilipolytic effect of insulin, that is, increased insulin sensitivity at the adipose tissue level.

Betaine addition did not affect the insulin antilipolytic effect in β-stimulated adipose tissue explants, independently of temperature (Figure 3). This is in agreement with in vivo studies showing no change in serum insulin, glucose and indices of insulin resistance in betaine-fed Iberian pigs under TN [25].

The results presented in the present study represent novel information that will help to better understand the interactions between heat stress and betaine regarding lipolysis in rustic breeds. Although the ex vivo approach allows the control of a number of variables in the fat cell environment, the influence of circulatory and nervous factors, active in situ, is impossible to evaluate. Therefore, the results obtained ex vivo require in vivo validation whenever possible. Definitely, questions must be raised about what conditions best represent the in vivo environment. Additionally, the long-term effects of heat stress and betaine on lipolysis would help to have a better perspective of lipid metabolism. It would also be interesting to extend the study to cosmopolitan pig breeds less resilient to HS than Iberian pigs.

## 5. Conclusions

Heat stress significantly decreases lipolytic response in the Iberian pig, promoting adipose tissue accretion. The addition of betaine may overcome this inhibition, suggesting that betaine supplementation may serve as a viable nutritional strategy to counteract the negative effects of heat stress on lipid metabolism. Future research should focus on elucidating breed-specific responses to betaine under varying environmental conditions to increase pig heat tolerance. All these insights together could help to optimize precision nutrition, improving the resilience of animals in heat-vulnerable regions.

## Data Availability

The data presented in this study are available on request from the corresponding author.
